# Atrio‐ventricular block and takotsubo syndrome: A review illustrated with two case reports

**DOI:** 10.1002/ccr3.5417

**Published:** 2022-02-15

**Authors:** Georgios Vavilis, Shams Y‐Hassan

**Affiliations:** ^1^ Departement of Medicine Karolinska Institute Huddinge, Stockholm Sweden; ^2^ Coronary Artery Disease Area Heart and Vascular Theme Karolinska University Hospital Stockholm Sweden

**Keywords:** AV‐block, broken heart syndrome, neurogenic stunned myocardium, pacemaker, takotsubo

## Abstract

Takotsubo syndrome (TS) can be complicated by life‐threatening arrhythmias. Data on the association of AV‐block, pacemaker implantation, and TS are scarce. We describe two cases of AV‐block associated with TS. AV‐block persisted despite the recovery of left ventricular dysfunction during follow‐up. A review of AV‐block and TS association is provided.

## Graphical abstract

Atrio‐ventricular block (AV‐Block) and Takotsubo syndrome (TS): A review illustrated with two case reports.

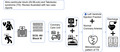

## 1 INTRODUCTION

Takotsubo syndrome (TS), known as broken heart syndrome, is a recognized acute cardiac disease entity.^1^ The term takotsubo (tako = octopus, tsubo=a pot) was introduced by Sato and Dote in 1990s to describe the left ventricular silhouette during systole in 5 patients presenting with clinical features of myocardial infarction but without obstructive coronary artery disease.^2^ The syndrome has a clinical and electrocardiographic presentation resembling that of an acute coronary syndrome (ACS).^3^ The defining feature of TS is the regional left ventricular wall motion abnormality (LVWMA) with a peculiar circumferential pattern resulting in a conspicuous ballooning of the left ventricle during systole.^4^ The syndrome may be complicated by heart failure, pulmonary edema, cardiogenic shock, left ventricular outlet tract obstruction, mitral regurgitation, life‐threatening arrhythmias, atrioventricular (AV) block, cardiac arrest, cardiac rupture, and death.^5^ Innumerable physical factors including sick sinus syndrome and AV‐block have been reported to trigger TS.^6^ Herein, we describe two cases where AV‐block was associated with mid‐ventricular pattern of TS. In both cases, the AV‐block persisted after pacemaker implantation and recovery of left ventricular dysfunction. Furthermore, the association and the cause‐and‐effect relationship between AV‐block and TS are discussed.

## 2 CASE PRESENTATIONS

### 2.1 Case history 1

An 85‐year‐old woman was referred to a tertiary center for further evaluation and treatment of suspected AV‐block III, congestive heart failure, and systemic arterial hypertension. The patient had a history of breast cancer treated with mastectomy. She was a smoker and had essential hypertension.

**FIGURE 1:**
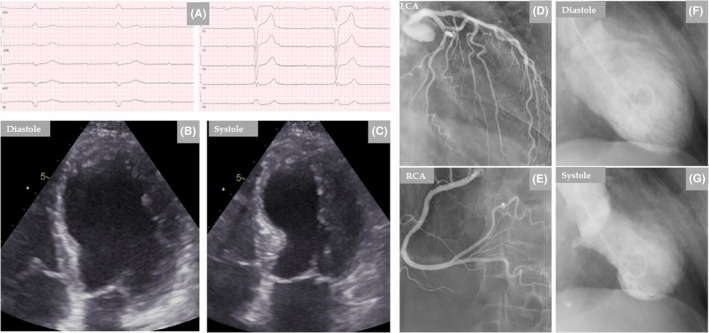
Twelve‐lead electrocardiogram (ECG) reveals high degree of AV‐block (A). TTE shows akinesia in the middle segments of the LV causing mid‐ventricular ballooning (B, diastole and C, systole). LCA and RCA are normal (D and E). Left ventriculography during diastole (F) and systole (G) confirms the echocardiographic findings of left mid‐ventricular ballooning consistent with mid‐ventricular pattern of TS

On physical examination, she had bradycardia 30/min, the respiratory rate was 14 brearhs/min and the blood pressure was high (244/155 mmHg). The patient apeared tired. The heart sounds were low with irregular rhythm and no murmurs. The lung ausculatation revealed diffuse crackles in the lungs and there were ankle oedemas bilaterally. Electrocardiogram showed AV‐block III with a ventricular rate of 33/min (Figure 1) and left bundle branch block (LBBB) without changes of ST‐segment or T‐waves. Plasma troponin T was moderately elevated at 777 ng/L (normal range <15), the NT‐pro‐BNP level was high at 4420 ng/ml (normal range <222).

The patient was transferred to the coronary care unit with continuous infusion of isoprenaline while waiting for a permanent transvenous pacemaker implantation. She also received treatment for symptomatic heart failure.

#### 2.1.1 Investigations and treatment

Transthoracic echocardiography (TTE) revealed moderate left ventricular dysfunction, with an estimated left ventricle ejection fraction (LVEF) 35%–40% and severe mid‐ventricular hypokinesis without evidence of mural thrombus (Figure 1B, C). The right ventricle and all valves were normal. Invasive coronary angiography revealed only mild atheromatous changes in the left (LCA) and the right coronary arteries (RCA) but no coronary stenoses or occlusions (Figure 1D, E). Contrast left ventriculography showed marked hypokinesia in the middle segments of the LV with good contractility in the apical and basal segments, giving findings consistent with mid‐ventricular TS (Figure 1F, G). A decision of double chamber permanent pacemaker implantation was taken, and it was inserted without complications. There was significant clinical improvement over a period of 2 days. She was discharged on Day 3 and returned home with in‐home services.

Five days after the discharge, the patient was readmitted due to shortness of breath and nocturnal dyspnea but no chest pain. On physical examination, she had clinical signs of heart failure. The heart rate was 81 beats per minute, the blood pressure was 193/105 mmHg, the respiratory rate was 30/min, and the oxygen saturation was 87% while she was breathing ambient air. New plasma troponin T level was 118 ng/L, the NT‐pro‐BNP level 9220 ng/ml. Electrocardiography showed pacemaker rhythm of 85/minute. Bedside TTE revealed persistent mid‐ventricular TS. The estimated LVEF was 35%–40%. Conventional treatment of heart failure was continued with clinical improvement. She was discharged on Day 6 receiving beta‐blockers, ACE inhibitors, and mineralocorticoid antagonist.

#### 2.1.2 Outcome and follow‐up

**FIGURE 2:**
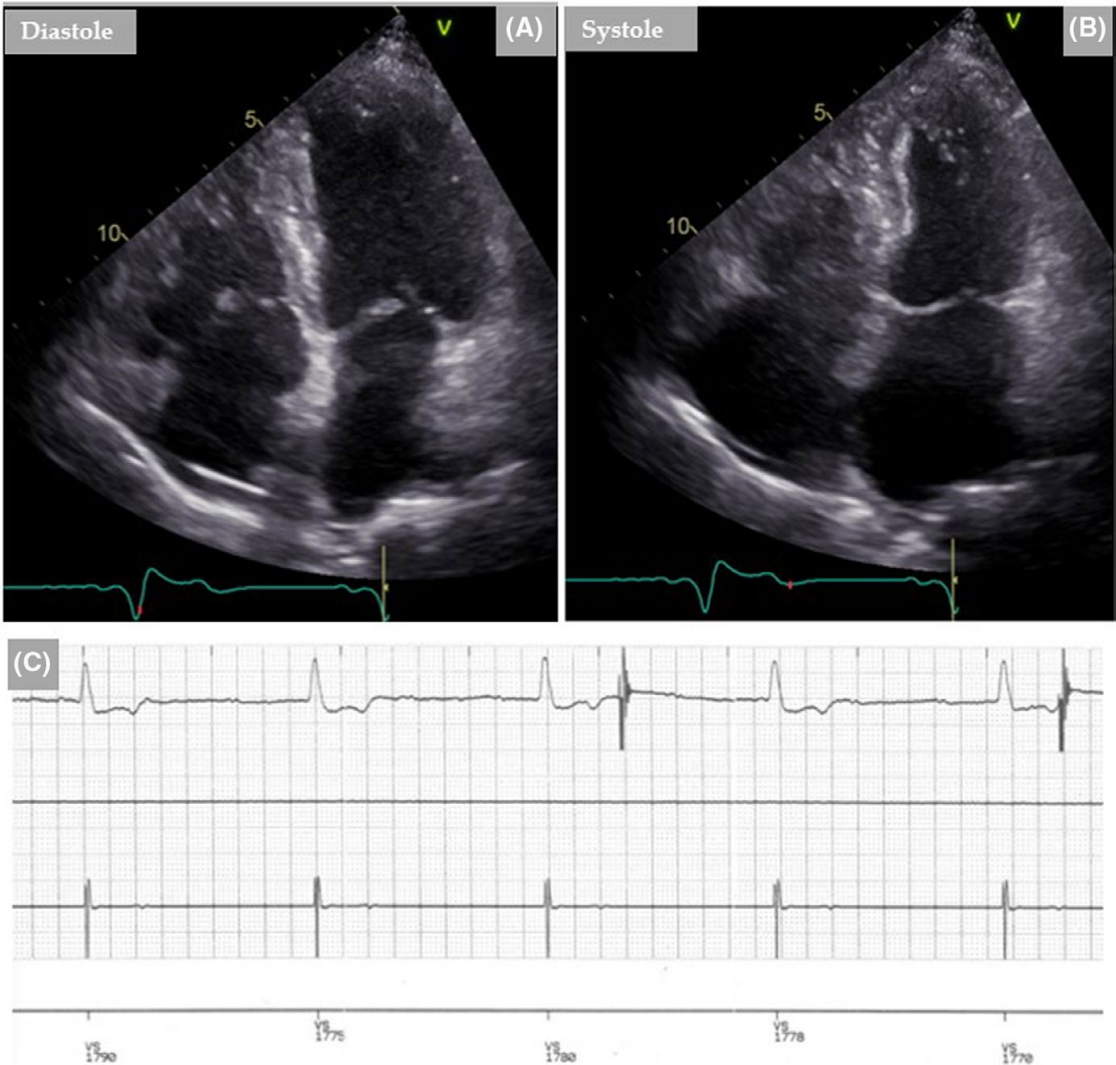
Follow‐up with TTE 3 months after the admission shows regress of TS and normalization of LVEF (A, diastole and B, systole). Endocardial ECG from the pacemaker interrogation one year later, shows persistent AV‐Block III (C)

The clinical course of the patient was determined during an outpatient control, three months after the first admission. A TTE revealed normalization of LVEF (Figure 2A, B). Pacemaker interrogation showed ongoing high‐degree AV‐block (Figure 2C) with the need for permanent ventricular pacing (up to 100%) while the need for atrial pacing was 20%, despite complete recovery of left ventricular systolic function.

### 2.2 Case history 2

**FIGURE 3:**
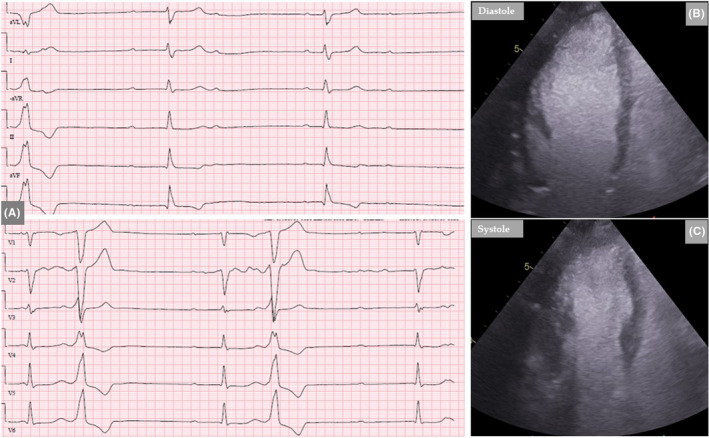
Twelve‐lead ECG reveals high degree AV‐block (A). Contrast TTE shows clear left mid‐ventricular ballooning (B, diastole and C, systole), a pattern consistent with TS

A 57‐year‐old woman presented to the emergency department with chest pain, dizziness, and bradycardia. Previously, she was an elite band ball player who continued to be active with several training passes in the week. She had a past‐history of smoking. A re‐elicited history confirmed significant emotional psychosocial stress. Two days before the admission, while the patient was on a walk, she developed suddenly stubbing substernal chest pain that was not radiated to the shoulders or jaw; it was accentuated during moderate physical exercise and resolved at rest. On admission day, she presented with dizziness. On physical examination, the patient appeared alert but frail. The pulse varied from 30‐35/minute and irregular, the blood pressure was 180/85 mmHg, and the remainder of the examination was normal. Electrocardiogram showed AV‐block III with a ventricular rate of 45/min (Figure 3A). The patient was transferred to the coronary care unit for further evaluation and a permanent transvenous pacemaker implantation. The plasma troponin T level was 253 ng/L and the ΝΤ‐pro BNP was 1510 ng/L. Results of other laboratory tests were normal. A contrast TTE showed segmental LV‐dysfunction involving the mid‐ventricular segments, resulting in conspicuous mid‐ventricular ballooning (Figure 3B, C). The estimated LVEF was 55%. There was mild mitral regurgitation, and the right ventricle was normal.

#### 2.2.1 Investigations and treatment

**FIGURE 4:**
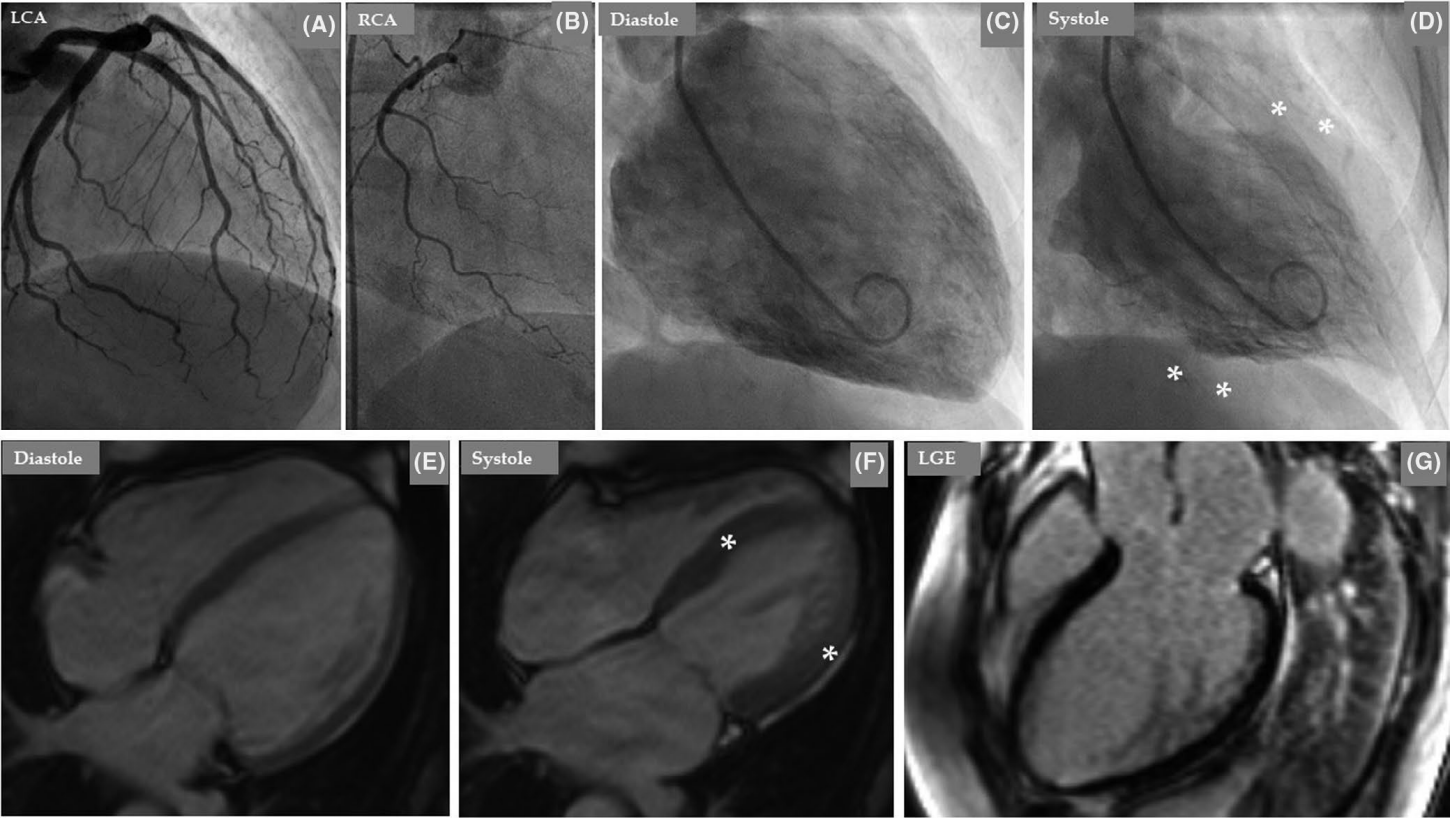
LCA (A) is dominant and normal; RCA (B) is hypoplastic and normal. Left ventriculography during diastole (C) and systole (D) shows left mid‐ventricular ballooning (D, white Asterix) consistent with mid‐ventricular pattern of TS. CMR imaging 6 days later showed improvement of LVEF (E, diastole and F, systole) but still mild hypokinesis in the middle segments of the left ventricle (F, white Asterix). There was no late gadolinium enhancement (LGE) in the left ventricular wall (G)

Invasive coronary angiography revealed normal dominant LCA and hypoplastic RCA (Figure 4A, B). Contrast left ventriculography showed hypokinesia in the middle segments of the LV with good contractility in the apical and basal segments, findings consistent with mid‐ventricular pattern of TS (Figure 4C, D). Cardiac magnetic resonance (CMR) imaging 6 days later revealed mild hypokinesia in the middle segments of the left ventricle with god contractions in the apical and basal segments with mildly reduced LVEF of 51% (Figure 4E, and F). Right ventricular function was normal. Native T1 imaging did not reveal any clear evidence of myocardial edema. Findings suggestive of myocarditis or a myocardial scar or other granulomatous diseases could not be detected (Figure 4G). The cardiac imaging study findings are consistent with mid‐ventricular pattern of TS. A double chamber permanent pacemaker implantation was performed on the seventh day of presentation.

#### 2.2.2 Outcome and follow‐up

**FIGURE 5:**
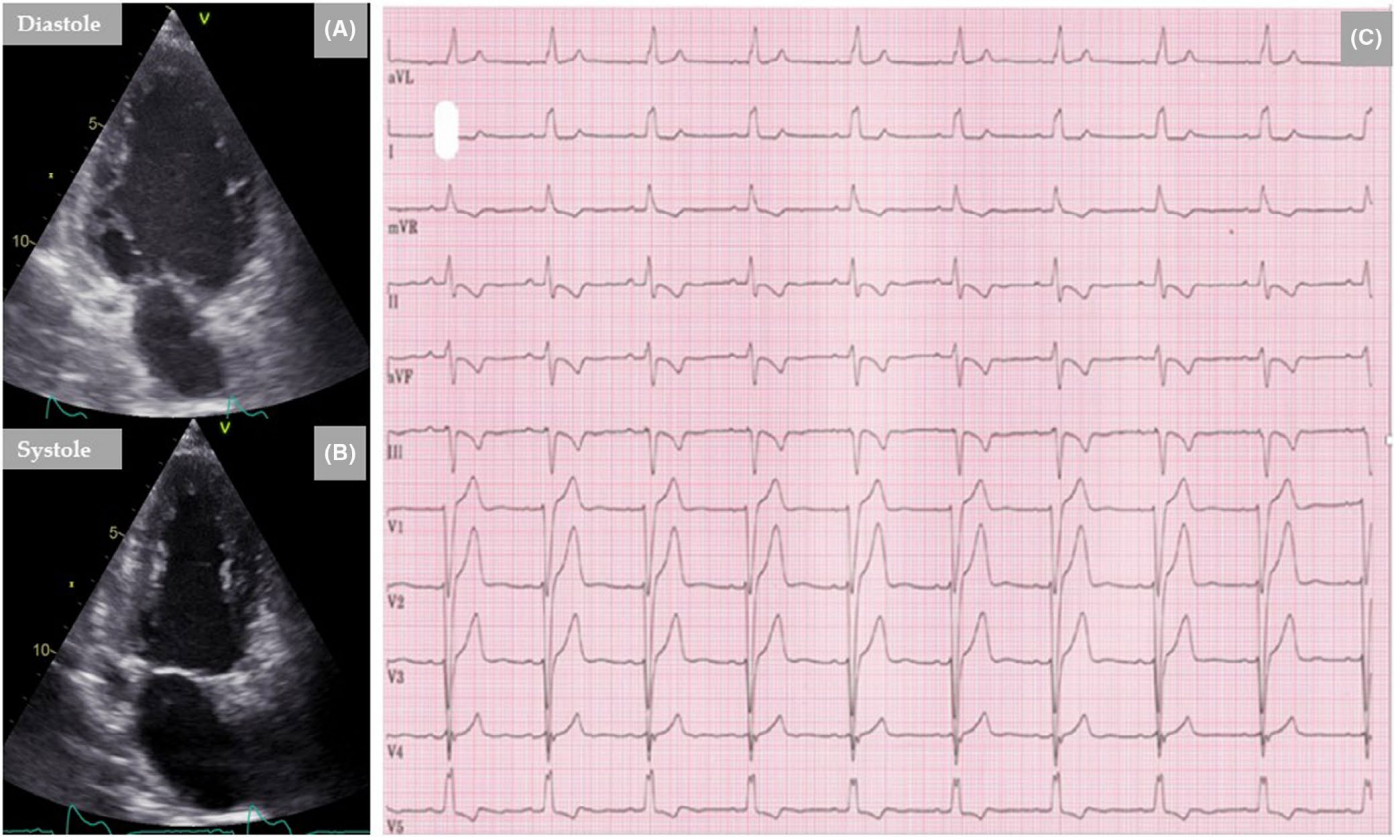
Follow‐up TTE 3 months later after the admission showing regress of TS and normalization of LVEF (A, diastole and B, systole). ECG from the pacemaker interrogation 3.5 years later showing restoration of sinus rhythm (C)

At three months of follow‐up, TTE revealed complete normalization of LV‐function (Figure 5A, B). Pacemaker interrogation showed 20% atrial pacing and 99% ventricular paced rhythm. At 12 months follow‐up, pacemaker interrogation confirmed AV‐block II type II as underlying rhythm, 12% atrial pacing and 100% ventricular paced rhythm. The last device controls at 3.5 years follow‐up showed intrinsic AV conduction at a ventricular rate 56 beats/min with LBBB (Figure 5C). This probably demonstrates an eventually resolution of the high degree AV‐block in more than 3 years after the index presentation.

## 3 DISCUSSION

The main findings in the two current cases, based on more than three‐years follow‐up time, are 1) both patients had advanced AV‐block and mid‐ventricular pattern of TS sparing the basal and the apical segments of the LV, 2) The AV‐block persisted despite the resolution of the LV‐dysfunction. The association of sinus bradycardia, sick sinus syndrome, AV conduction defects, and TS have been reported.^6^, ^7^ In a review of 25 case series constituting of 816 patients with TS reporting on arrhythmias, Syed et al.^8^ reported sinus node dysfunction in 1.3% and AV‐node dysfunction in 2.9%. Stiermaier et al.^7^ reported complete AV‐block in 8 of 286 (2.8%) of patients with TS. The difference between our cases and previous ones is the long‐term follow‐up. It allowed us to confirm complete resolution of the LV‐dysfunction and continued AV‐block even one year after the index presentation.

### 3.1 AV‐block triggering TS/ AV‐block secondary to TS

Cases of AV‐block preceding or occurring in association with the onset of TS have been reported.^8^, ^9^, ^10^ The common features of all these cases are the apical or mid‐ventricular pattern of TS sparing the basal segments of the LV and the AV‐block persisted after complete resolution of the LV‐dysfunction, which argues for the fact that AV‐block triggered TS just like the 2 cases we have described. The absence of an obvious acute physical or emotional trigger factor apart from the AV‐block and the symptoms attributable to AV‐block in our patients resulted in the conclusion that the AV‐block was the most probable physical factor that triggered TS in our patients.

TS may be complicated by transient but prolonged AV‐block. Cases with TS and transient AV‐block treated by temporary pacemaker implantation have been reported.^5^, ^6^, ^7^, ^8^, ^9^, ^10^, ^11^, ^12^, ^13^ A case of TS and AV‐block where AV‐block persisted for 2 weeks resulting in permanent pacemaker implantation.^13^ At 1‐month follow‐up, the LV‐function recovered and there was no AV‐block. This implies delayed recovery of AV‐block which was most probably secondary to TS.

### 3.2 Cause‐and‐effect relation between AV‐block and TS

TS is an acute cardiac syndrome, which is usually preceded by a trigger factor in about 70% of cases.^4^ TS is also associated with relatively high complication rates as heart failure, cardiogenic shock, tachy‐ and brady‐arrhythmias, and many others.^4^ The most confounding factor is when the trigger factor for TS development is of cardiac origin. ACS is still regarded by some investigators as an exclusion criterion for TS. ACS is one of the trigger factors for TS development and TS may be complicated by ACS.^14^ The same problem is applied to the association of AV‐block and TS. In some cases, the cause‐and‐effect relationship in patients with TS and AV‐block is difficult to explain. In others, the chronological sequence may be apparent. Most of the reported cases, where AV‐block was associated with TS, revealed that either AV‐block preceded TS or the AV‐block persisted despite complete recovery of LVWMA. This indicates that most probably AV‐block was the trigger factor for TS. As noted above, almost all reported cases of TS and AV‐block were of apical or mid‐ventricular pattern sparing the basal segments of the left ventricle. In fact, the occurrence of AV‐block in the apical or mid‐ventricular patterns of TS with sparing of the basal segments is hard to be explained by TS because the myocardium adjacent and around the AV‐node is spared. In addition, the increases of sympathetic tone challenges AV‐block as a complication of TS. In a study on 286 patients with TS, Stiermaier et al.^5^ reported a prevalence of complete AV‐block in 2.8% (*n* = 8). Seven patients received permanent pacemaker because of complete AV‐block (*n* = 6) or sinoatrial block (*n* = 1). Regular device check‐ups were available in 2 patients and demonstrated ongoing high‐degree AV‐block despite resolution of the LV‐dysfunction. Interestingly, three patients with transient brady‐arrhythmias who did not receive devices died shortly after hospital discharge from unknown causes. The authors concluded that brady‐arrhythmias in the acute setting of TS may require permanent pacemaker implantation. Worth to mention is that 13 of 286 (4,5%) studied patients already had a pre‐existing permanent pacemaker at the time of admission for the following reasons: complete AV‐block (*n* = 6), sick sinus syndrome (*n* = 4), and bradycardia–tachycardia syndrome (*n* = 3). However, there are case reports indicating that AV‐block may have been a complication of TS and the AV‐block has disappeared after recovery of the LVWMA.^11^ Transient edema of the stunned myocardium, microvascular compression caused the contracted stunned myocardium^15^ or an increase in vagal tone may explain the transient AV‐block secondary to TS.

## 4 CONCLUSION

Two cases of TS with mid‐ventricular ballooning triggered by AV‐block are described. In both cases, the AV‐block persisted after pacemaker implantation and complete recovery of LV‐dysfunction. Furthermore, the association of AV‐block, pacemaker implantation, pacemaker dysfunction, and TS including the cause‐and‐effect relationship between heart‐block and TS are briefly reviewed.

## CONFLICT OF INTEREST

None declared.

## AUTHOR CONTRIBUTIONS

Both authors have equally contributed in the collection of data and writing of this paper. Furthermore, both co‐authors were clinically involved in the clinical management of both cases.

## CONSENT

The authors confirm that written consent for submission and publication of this case report, including images, and associated text, has been obtained from the patients in line with the journal’ s patient consent policy.

## Supporting information

Supplementary Material

## Data Availability

The authors declare that all supporting data are available within the article and Supplementary Materials.
