# Supplementary material for: Atrio‐ventricular block and takotsubo syndrome: A review illustrated with two case reports
Source: Clin Case Rep. 2022 Feb 15;10(2):e05417. doi: 10.1002/ccr3.5417 (PMC8847407; doi:10.1002/ccr3.5417)
Supplement: Supplementary file 1 — Supplementary Material [file CCR3-10-e05417-s001.docx]

**Learning points**

High-degree AV-block, associated with acute mid-ventricular pattern of takotsubo syndrome (TS) persisted after pacemaker implantation and complete recovery of left ventricular function during follow up. Clinicians should be aware about the cause-and-effect relationship between AV-block and TS and should consider implantable pacemaker device in some patients before discharge to improve the clinical outcome
